# Transcytosis - An effective targeting strategy that is complementary to “EPR effect” for pancreatic cancer nano drug delivery

**DOI:** 10.7150/thno.38587

**Published:** 2019-10-17

**Authors:** Xiangsheng Liu, Jinhong Jiang, Huan Meng

**Affiliations:** 1 Division of Nanomedicine, Department of Medicine, University of California, Los Angeles, California 90095, United States; 2 California NanoSystems Institute, University of California, Los Angeles, California 90095, United States

**Keywords:** Transcytosis, EPR effect, nano drug delivery

## Abstract

Numerous nano drug delivery systems have been developed for preclinical cancer research in the past 15 years with the hope for a fundamental change in oncology. The robust nanotherapeutic research has yielded early-stage clinical products as exemplified by the FDA-approved nano formulations (Abraxane^®^ for paclitaxel and Onyvide^®^ for irinotecan) for the treatment of solid tumors, including pancreatic ductal adenocarcinoma (PDAC). It is generally believed that enhanced permeability and retention (EPR) plays a key role in nanocarriers' accumulation in preclinical tumor models and is a clinically relevant phenomenon in certain cancer types. However, use of EPR effect as an across-the-board explanation for nanoparticle tumor access is likely over-simplified, particularly in the stroma rich solid tumors such as PDAC. Recently, ample evidences including our own data showed that it is possible to use transcytosis as a major mechanism for PDAC drug delivery. In this mini-review, we summarize the key studies that discuss how transcytosis can be employed to enhance EPR effect in PDAC, and potentially, other cancer malignancies. We also mentioned other vasculature engineering approaches that work beyond the classic EPR effect.

## Introduction

**Figure 1:**
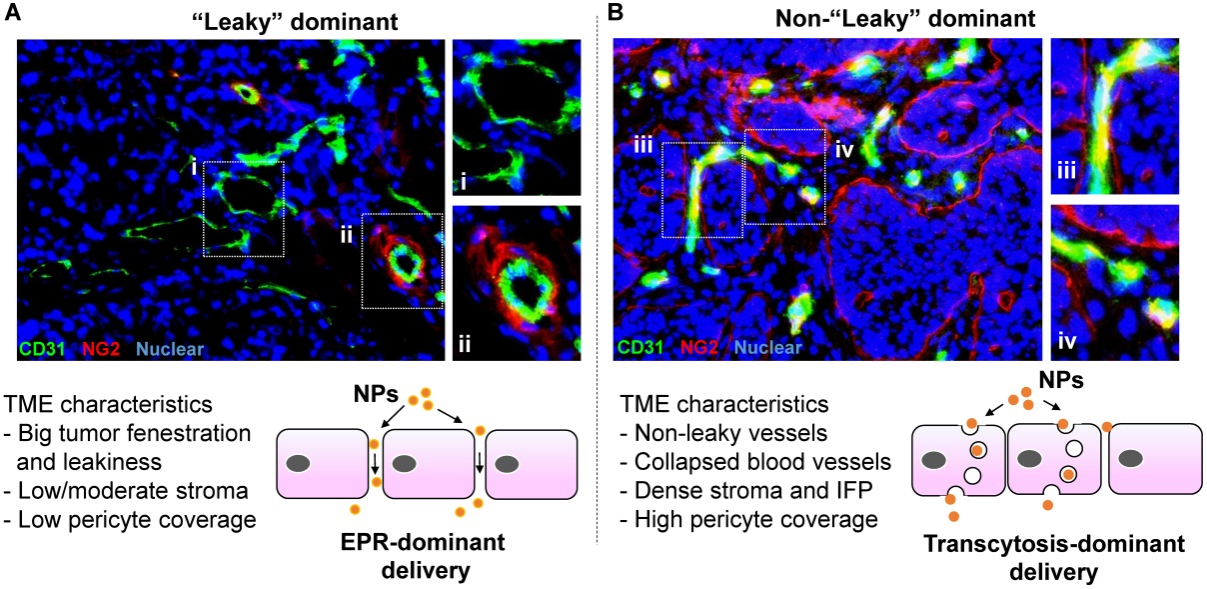
** Representative solid tumor IHC staining to show “leaky” vs “non-leaky” tumor types.** Human MCF-7 breast cancer (A) and BxPC3 pancreatic cancer (B) tissues were retrieved from our historical samples and OCT embedded for frozen section. Two-color immunohistochemistry staining was performed. The endothelial cell marker (CD31) was labeled in green (FITC), and the pericyte marker (NG2) was labeled in red (Alexa Fluor 594). Nuclear was labelled by Hoechst dye. Zoom in pictures to show the extent of pericyte coverage in each tumor type. While the blood vessels in MCF-7 tumor exhibit low pericyte coverage and round-like structure, we frequently observed structurally collapsed or obstructed blood vessels in BxPC3 tumor, which also contain high pericyte coverage. Accordingly, we assigned MCF-7 and BxPC3 tumors into “leaky” and “non-leaky” categories, respectively. The important tumor microenvironment (TME) characteristics are summarized. In our opinion, while EPR effect may play a key role in the “leaky” tumor (A, lower panel), transcytosis becomes more important in the “non-leaky” tumor type (B, lower panel).

Most nanocarriers currently being tested in clinical trials rely on passive delivery, which conceptually depends on the enhanced permeability and retention (EPR) effect^1^, ^2^. While, the EPR effect is often explained as the presence of “leaky” tumor vasculature, the size-controlled delivery of nanoparticles varies dramatically among different cancer types^3^-^5^. In cancer patients, it is encouraging to see the incremental evidences of EPR using liposome and polymeric nanocarriers, which suggests that EPR is a clinically relevant phenomenon in certain cancer types or patient populations^3^. However, another suggestion is that the abnormal vascular fenestrations and leakiness of highly vascular human xenografts in mice may represent an experimental artifact^6^, ^7^. Accordingly, “EPR effect in patients” and “targeting principle beyond EPR” become “hot” topics for research and translational nanomedicine study. In fact, there is a major debate on the effectiveness of EPR effect in cancer animal models and ultimately patients^3^, ^6^, ^8^. In a meta-analysis based on 10 years nanomedicine literature, the authors concluded that ~0.7% (median) of the injected nanoparticle dose accumulated in solid tumors^9^. Other experts indicated that the EPR effect of 0.7% was an unfair and unconventional calculation in determining nanomedicine tumor homing efficiency^10^. A recent literature further pointed out the key aspects that were overlooked in the aforementioned meta-analysis, such as sophisticated nanocarrier physicochemical properties, experimental errors from unstable particle labeling, neglected contributions from circulation half-life, tumor type, weight, interstitial fluid pressure (IFP), etc^8^, ^10^. In the setting of solid tumors with a thick dysplastic stroma, the putative EPR effect is governed by the factors that are way beyond enlarged tumor fenestrations because these tumor types (including PDAC) may develop vasculature that are structurally collapsed or obstructed by tumor-associated fibroblasts or pericytes that adhere tightly to the endothelial cells (Figure 1). The cellular and non-cellular heterogeneity in the tumor microenvironment (TME), which is incompletely understood, collectively determines the fate of nanoparticle tumor access. In this manuscript, we mainly focus on nano drug delivery in PDAC with a view to discuss transcytosis-mediated tumor targeting, an EPR-independent targeting approach without the need of tumor vasculature leakiness.

PDAC is the 4^th^ leading cause of cancer death. According to the American Cancer Society's estimation, there were 56,770 new PDAC cases and 45,750 patient death cases in the US in 2019^11^, ^12^. This accounts for approximately 3% of all cancer cases in the US and about 7% of all cancer deaths^11^, ^12^. Unlike other cancer malignancies, chemotherapy is the most frequently used treatment for the majority PDAC patients because of the late diagnosis that leads to advanced disease. At this stage, the standard treatment includes gemcitabine (GEM) and classic or modified FOLFIRINOX (a 4-drug regimen). Recently, the FDA approved the use of GEM plus Albumin-bound paclitaxel nanocomplex (Abraxane) and liposomal irinotecan nanocarrier (Onivyde) plus fluorouracil for PDAC treatment, which lead to an improvement of overall survival of ~2 months^13^, ^14^. The lack of a robust response to these first-generation nano formulations is in part due to the unfavorable PDAC TME that prevents drug delivery. In addition to cancer cells, a major pathological feature is the presence of thick tumor stroma, which lowers the putative EPR effect and nanoparticle access. In PDAC, the stroma barrier contains a variety of cellular and non-cellular components, such as pericytes, endothelial cells, fibroblasts, tumor associate macrophage, T cells, NK cells, collagen deposition, etc^15^, ^16^. The complexity in the TME also comes from the biophysical components, such as acidity, hypoxia, high tumor IFP, etc.^15^, ^16^. Collectively, fibrotic stroma and abnormal vasculature negatively impact drug delivery in PDAC.

Instead of relying the enlarged tumor fenestration that may not dominate in PDAC, we are asking how the nanoparticles can cross PDAC vasculature. This turns out to be a technically challenging question to answer for multiple reasons. First, due to logistic limitation, most preclinical studies rely on observations at empirically selected late time points. This experimental design leads to “endpoint” observations, which answer the question about whether the injected nanoparticle can reach the tumor site, but not necessarily how the nanoparticles reach the tumor site. Second, ultrastructural visualization of nano particulates in heterogeneous TME requires high operational standards in terms of sample preparation, imaging condition and equipment status. By considering these challenges, it would be ideal to make an *in situ* observation with ultrastructural resolution to answer the question about if the enlarged tumor fenestration is the solo factor that determines nanoparticle tumor access. Alternatively stated, does nanoparticle extravasation primarily rely on “leakiness” (Figure 1A, lower panel) or there is other mechanism that operates complementarily, *i.e.* through a transendothelial transport pathway (*a.k.a.* transcytosis) that assists nanoparticle tumor homing effectiveness (Figure 1B, lower panel).

**Figure 2:**
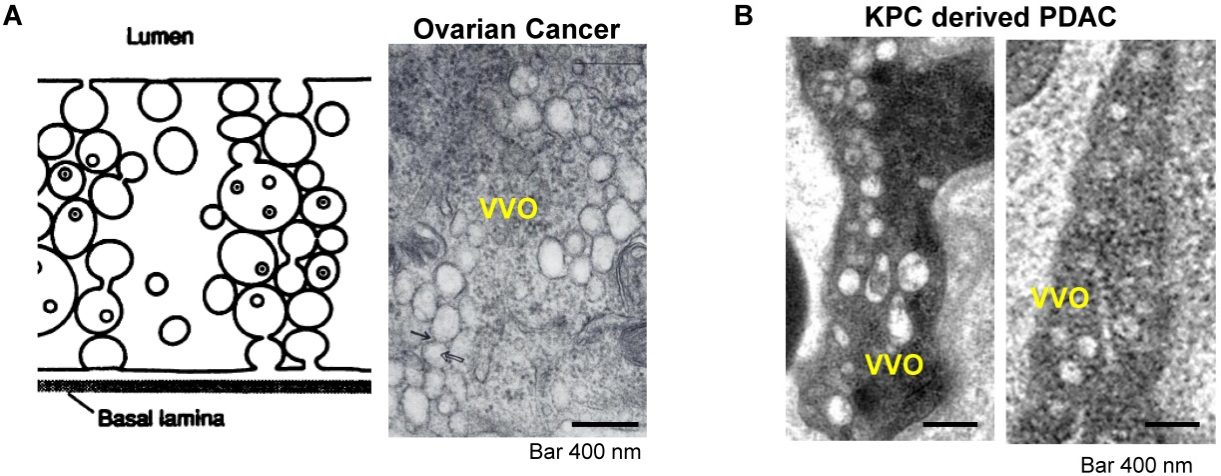
** Transcytosis and vesiculo-vacuolar organelle (VVO).** (A) Left: Schematic of VVO mediated transcytosis pathway; right: ultrastructural TEM view shows VVOs to consist of grape-like clusters of interconnecting vesicles and vacuoles in abluminal in a subcutaneous mouse ovarian tumor. Adapted with permission from ref.^17^. (B) Ultrastructural TEM shows the VVOs structures in an orthotopic KPC-derived PDAC tumor. Adapted with permission from ref.^20^.

**Figure 3:**
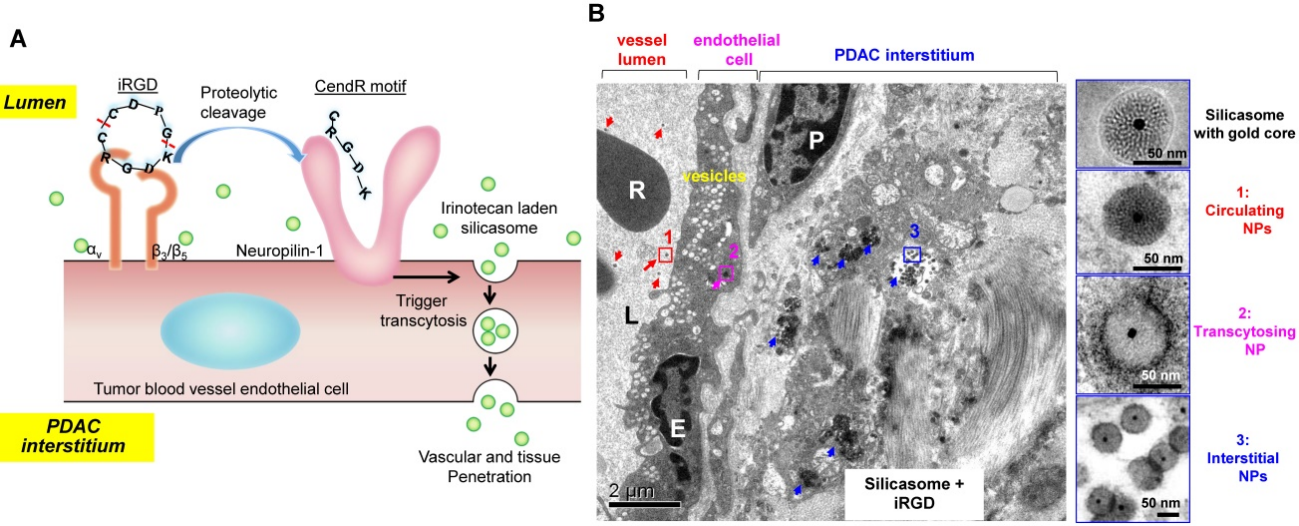
(A) Schematic of the iRGD-mediated transcytosis mechanism for silicasome nanocarrier delivery in PDAC tumor. (B) Ultrastructural TEM views show iRGD co-administration mediated silicasome transcytosis process in orthotopic KPC tumor. The TEM image shows gold core labeled silicasomes in (i) the lumen of a tumor blood vessel (red arrows), (ii) transport in the endothelial vesicles (pink arrow), and (iii) deposition in the tumor interstitium (blue arrows). High-magnification images of regions 1 through 3 are provided in the panels on the right. E, endothelial cell; P, pericyte. Scale bar: 2 μm (left panel); 50 nm (right panels). Adapted with permission from ref.^20^.

A very interesting observation that appeared in old ultrastructural literatures was the demonstration that ovarian tumor vascular endothelial cells displayed a network of tubular vesicles (*a.k.a.* the vesico-vacuolar organelle or VVO) (Figure 2A)^17^-^19^. VVOs were described as “grape-like” clusters of interconnecting vesicles and vacuoles, which span the entire thickness of vascular endothelium^17^. The authors suggested that these VVOs may provide a potential trans-endothelial path between the vascular lumen and the extravascular space, facilitating macromolecule transcytosis even without the need of vasculature leakiness^17^. Our own observation showed that VVO-like structures can be found in an orthotopic PDAC model using Kras mutant PDAC cells (derived from a spontaneous PDAC tumor from a transgenic *Kras^LSL-G12D/+^Trp53^LSL-R172H/+^Pdx1-Cre* mouse) (Figure 2B)^20^. Previous studies also showed an endocytic transcytosis pathway that can be therapeutically elevated by tumor-penetrating iRGD peptides (CRGD[K/R]GP[D/E]C)^21^, ^22^. iRGD is capable of homing to the tumor-specific integrins expressed on the endothelial cells on tumor vasculature (but not normal cells)^21^, ^22^. In iRGD, the CendR motif is not C-terminal, but an active CendR motif that can be generated through proteolytic cleavage (Figure 3A)^23^, ^24^. The exposed CendR motif interacts with a multifunctional, VEGF-binding, non-tyrosine kinase receptor, neuropilin-1 (NRP-1). NRP-1 binding triggers a mass transcytosis pathway that mimics macropinocytosis (except that NRP-1 receptor is involved), and is similar in concept to the VVO's^25^-^27^. Moreover, NRP-1 expression correlates with tumor progression and poor prognosis in various cancers, including PDAC^27^. Accordingly, the iRGD peptide is capable of promoting the penetration and tumor cell entry of a wide range of therapeutics in tumor models, including BxPC-3 and PC-09 PDAC models^27^. The therapeutics includes free drugs, macromolecules (dextran), dyes (Evans blue), peptide, antibodies, liposomes, and Abraxane^21^, ^22^, ^27^, ^28^. Recently, we showed that the anti-cancer efficacy of an irinotecan loaded silicasome nanocarrier can be significantly improved by the co-administration of free iRGD peptide even without the requirement of covalent attachment^20^. This led to a ~4-fold nanoparticle uptake increase at the orthotopic KPC PDAC site, leading to enhanced efficacy at primary and metastatic sites. Moreover, through the use of transmission electron microscopy (TEM), we obtained ultrastructural *in situ* evidence showing the appearance of grouped vesicles in PDAC endothelial cells, with the ability to carry gold nanoparticle labeled silicasomes from the blood vessel lumen to the PDAC matrix, without the requirement of tumor fenestration (Figure 3B)^20^.

In addition, it was also demonstrated that albumin can mediate a transcytosis process in endothelial cells by targeting the 60-kDa glycoprotein (gp60) receptor, which binds to caveolin-1 by forming transcytotic vesicles^29^-^31^. Based on this caveolae-mediated transcytosis mechanism, many albumin-based drug delivery nanoparticles were developed for cancer treatment including PDAC^32^-^36^. One well-known example is the albumin-bound nanoparticle (nab) paclitaxel (Abraxane^®^), which achieved efficient delivery of the drug to the PDAC and other tumor sites by taking the advantage of caveolae-mediated transcytosis mechanism^35^, ^37^. Besides the albumin, another impressive example of receptor-mediated transcytosis of nanoparticles in PDAC is the development of urokinase plasminogen activator receptor (uPAR) targeting imaging nanoparticles^38^. The association of uPAR with caveolae first facilitates nanoparticles crossing the endothelium *via* transcytosis. This allows the particle entry into perivascular tumor areas, followed by the binding to uPAR-expressing PDAC tumor cells and tumor-associated stromal cells^37^. This approach has resulted in highly selective delivery of imaging probes into primary and metastatic pancreatic cancer lesions^38^. In addition to surface modification, recent data suggested that surface charge may also play an important role during the transcytosis process^39^-^43^. A macropinocytosis-mediated transcytosis was observed in cationic nanoparticles which exhibit efficient tumor penetration when compared to the treatment using anionic and neutral particles^39^-^42^. Zhou *et al*., designed a γ-glutamyl transpeptidase-responsive camptothecin-polymer conjugate. The idea was to use γ-glutamyl transpeptidase that was overexpressed on the endothelial cell membrane to cleave the γ-glutamyl moieties, leading to the positive primary amines on the particle surface^43^. The resulting cationic conjugate actively infiltrated throughout the tumor tissue through caveolae-mediated (not macropincytosis) endocytosis and transcytosis, which enable deep tumor penetration with effective anticancer drug delivery^43^. A brief summary of transcytosis mediated nanocarrier delivery examples in PDAC was provided in Table 1.

Transcytosis is a type of transcellular transport across the interior of a cell which extensively studied initially for macromolecules delivery through the blood-brain barrier (BBB)^30^, ^44^-^54^. Actually, transcytosis-based mechanism has been used to achieve efficient delivery of drugs and genes by nanocarriers to various tissues^55^-^58^. In addition to PDAC, transcytosis is also involved in variety of solid tumor cancers, such as breast cancer^33^, ^37^, ^42^, ^59^-^61^, lung cancer^37^, ^62^, prostate cancer^37^, ^42^, brain cancer^63^-^68^, melanoma^42^, ^58^, gastric cancer^69^, colorectal cancer^32^, ^33^, ^37^, ^41^, ^42^, ^69^ and other cancers^37^. One of the most recognized cases is drug delivery in brain cancers (e.g., glioma and glioblastoma) using transferrin (Tf) modification with the hope to across BBB^50^, ^68^, ^70^, ^71^. For example, Chang *et al.* demonstrated that Tf-coated PLGA-NPs entered massively within brain-developed F98 glioma tumors^64^. Porru *et al*. also developed zoledronic acid loaded nanoparticles with Tf-conjugation for transcytosis mediated BBB access and efficacy improvement in an orthotopic glioblastoma model^65^. Since tumor formation in brain may interfere BBB integrity, these observations, in our opinion, may be the combined effect of pathological vasculature leakiness and transcytosis-mediated nanoparticle brain access. Interestingly, Williams *et al.* found a type of PLGA-PEG mesoscale nanoparticles (~400 nm) selectively target to kidney via transcytosis mechanism across depend predominantly on size and surface functionalization (non-opsonizing surface) but is independent of moderate surface charges^72^. It becomes a very promising strategy of nanomedicines for kidney diseases^73^. Impressively, Leng *et al.* design a series of linear and branched histidine-lysine (HK) peptide carriers as nonviral vectors for gene and siRNA delivery, this type of HK polyplexes can target neuropilin-1 receptor on endothelial cells and tumor cells which mediated the transcytosis through the tumor endothelium and lead high tumor distribution and efficient transfection in MDA-MB-435 breast cancer model^74^, ^75^. The study further proved that the NRP-1 mediated transcytosis can be interfered by NRP-1 antibody blocking or enhanced using the approach of restricting nutrients with a glucose-transport inhibitor^74^.

While the use of transcytosis to improve drug delivery is still not fully understood yet, ample evidences strongly suggested that physicochemical characteristics of nanocarriers may determine the effectiveness of transcytosis at tumor site. This includes the early-stage observations on particle chemical composition, size, shape, charge, surface modification, which may alter the rate and abundance of transcytosis^76^-^78^. However, it is too early to summarize a consensus to reproducibly activate transcytosis-mediated particle tumor access because these physicochemical parameters may collectively impact the process, which is further complicated by non-material factors, such as fibrotic status and IFP. Since favorable systemic circulation feature and intratumoral access may have different requirements with the respect to material properties, interesting strategies, such as the stimuli-responsive nanocarriers that may alter size/charge in circulation *vs* tumor have generated promising preclinical outcome^43^, ^76^, ^79^-^82^.

Another example to broaden the EPR concept is increased stromal vascular access by reducing pericyte coverage in PDAC^83^-^87^. While pericyte interference exhibits distinct mechanism as compared to transcytosis activation, it provides an alternative approach to improve drug delivery in non-leaky tumor, which may function beyond EPR effect. This challenge was met by designing a PEI/PEG-coated mesoporous silica nanoparticle that can be used for attaching a small molecule TGF-β receptor kinase inhibitor, LY364947^87^. This drug interferes with the dominant signaling pathway for pericyte recruitment and adherence to endothelial cells. This carrier was derived through iterative design to achieve monodisperse particles of optimal size (~50 nm), stable copolymer attachment, maximum colloidal stability, and biocompatible polymer size selection (e.g., PEI 1.8 kD), and through choosing from a range of small molecule inhibitors to find a TGF-β inhibitor prototype with stable, pH-sensitive and high affinity binding. Ultimately, we obtained a nanocarrier containing high wt% LY364947. When tested in a human BxPC3 PDAC xenograft, this carrier could effectively interfere in pericyte adhesion to endothelial cells within 2 hours of intravenous injection. This allowed the development of a “two-wave” approach, in which the LY364947 nanocarrier was used as the 1^st^ wave to open the stromal vascular gate, thereby allowing rapid tumor entry by 2^nd^ wave drug carriers. This is in the line with nano-enabled engineered approach, which uses a multistage/multistep combination treatment to provide an impact on PDAC stroma, such as augmented blood vessel permeability, inhibition of drug inactivating enzymes and/or target specific biological factors^15^, ^88^-^91^.

**Figure 4:**
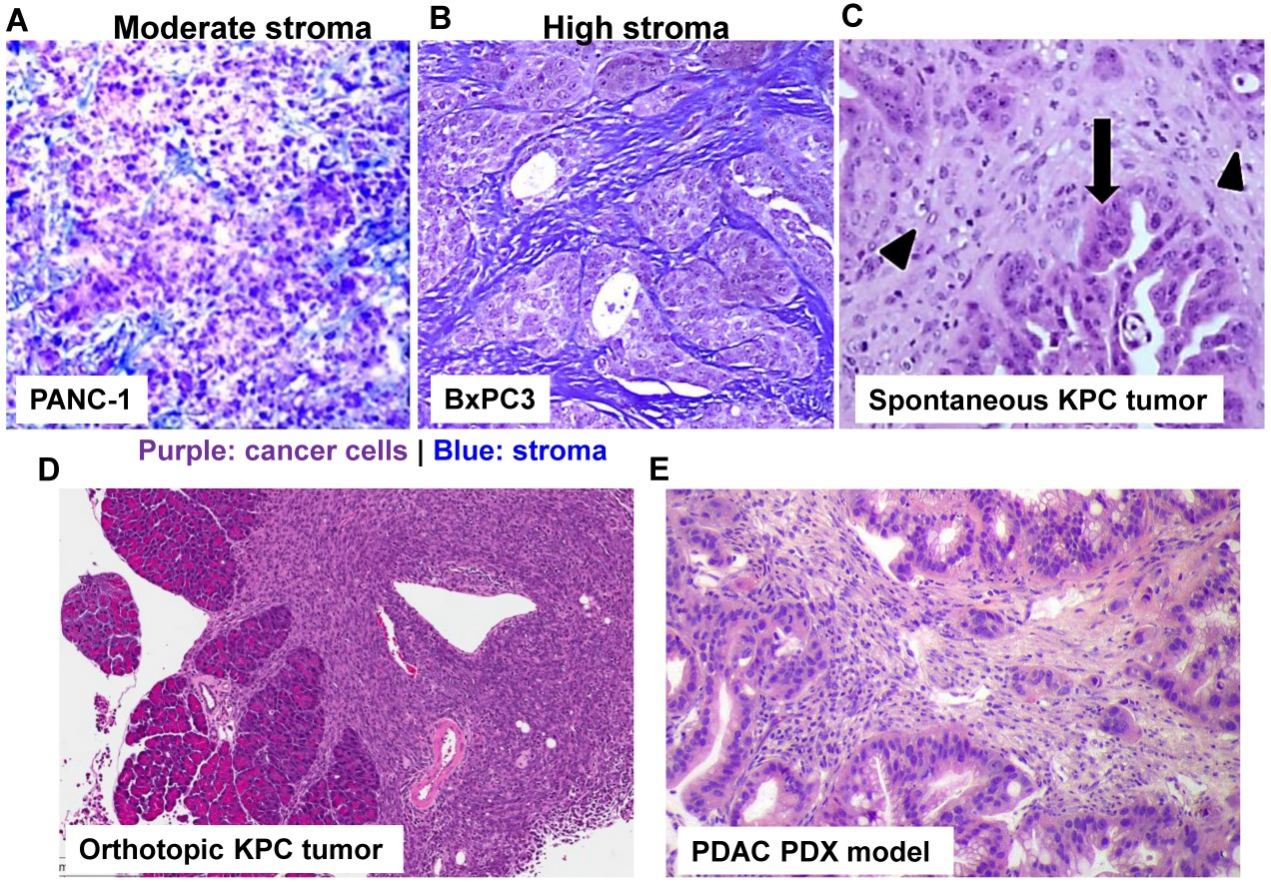
** Use of stringent PDAC cancer models to study drug delivery using nanoparticle.** With the rapid development of PDAC cancer biology, it has been possible by employing different PDAC mouse models to better understand the molecular mechanism underlying pancreatic cancer, including nanoparticle-mediated drug delivery. Trichrome staining of PANC-1 xenograft (A) and BxPC3 (B) in nude mice. While BxPC3 tumor (Kras WT) is usually regarded as stroma-rich, PANC-1 tumor (Kras mutated) contains moderate level of stroma content. With the recent success in the production of genetically engineered mouse models (GEMMs), it is theoretically possible to test nanotherapeutics in KPC model in which the conditional expression of mutant Kras^G12D^ and Trp53^R172H^ is governed by a pancreas-specific Cre. Without the involvement of Cre, a transcriptional and translational STOP cassette flanked by loxP sites silences the expression of mutant Kras^G12D^ and Trp53^R172H^. In KPC tumor (C, adapted with permission from ref.^93^), substantial nuclear abnormalities occur and glands appear embedded in the tumor stroma (arrowheads) with completely random organization (arrows). However, a major pitfall using spontaneous KPC model is the variable growth characteristics of the spontaneous KPC model and the number of animal experiments that can be undertaken. Therefore, the variable tumor development and unfavorable logistics, precludes widespread use of KPC model. In order to perform robust experiment, we have established immortalized luciferase-transfected cell lines derived from spontaneous KPC tumors, and have used them to establish a surgical procedure for orthotopic tumors in immunocompetent, syngeneic B6/129 mice (D). We have confirmed that orthotopic implant in the pancreas leads to predictable tumor development within 1-2 weeks and mimic human PDAC characteristics such as local invasion of the G.I.T. and liver metastases after 3-5 weeks. Moreover, the availability of PDAC PDX model (E) allows the study of patient-specific response and personalized nanomedicine.

From the translational nanomedicine aspect, there is a clear agenda to predict and quantify the EPR effect in cancer patients. This notion holds true to explore new mechanism(s) such as transcytosis to facilitate nanocarriers' tumor access, especially in non-leaky tumor types. It is noteworthy to dissect the complexity of each cancer indication as well as the differences among each individual with the same cancer indication. In fact, MRI imaging (using fluorescently labeled iron oxide nanoparticles) followed by quantitative intravital fluorescence visualization can identify “responder” for nanomedicine therapy^92^. To effectively discern the heterogeneous nanoparticle tumor access in preclinical study, it is critical to consider stringent tumor models as compared to data generation using the convenient but highly artificial subcutaneous xenograft model. It is generally agreed upon in the field that new therapies (which is true for PDAC nano therapeutics) should be tested in the advanced models (Figure 4), which closely mimics disease characteristics^93^. This includes primary human patient-derived xenograft (PDX) models, which can be implanted subcutaneously and orthotopically in NSG mice immediately after surgical resection^94^. In this regard, the selection of a tumor PDX pair with differential NRP-1 expression on the tumor vasculature demonstrated differences in carrier uptake and irinotecan delivery during iRGD-mediated transcytosis activation. Our data indicated that it is necessary to contemplate the usage of a personalized approach to PDAC chemotherapy to enhance the efficacy of the irinotecan silicasome carrier by iRGD co-administration^20^.

In summary, it is not surprising that major efforts are underway to study drug delivery using nano-enable approach for PDAC treatment. It becomes clear that transcytosis (perhaps with other unknown mechanisms) may coexist with the so-called EPR effect, which may not be a dominate factor in the case of PDAC. Further investigations are still required to fully understand this emerging approach, which may allow nanoparticle tumor targeting with major augment with respect to homing abundance, efficiency and time kinetics.

## Figures and Tables

**Table 1:** Examples of transcytosis mediated nanocarrier delivery in PDAC

Formulation	Transcytosis mechanism	Size	Zeta potential	Cancer model	Accumulation increased in tumor	ref
iRGD-conjugated lipid micelles	VVOs mediated	15-25 nm	n/a	Orthotopic human MIA PaCa-2 xenograft	n/a	22
Lipid coated mesoporous silica nanoparticle (silicasome) Co-delivery with free iRGD peptide	VVOs mediated	~130 nm	~ -10 mV	Orthotopic murine pancreatic KPC-derived tumor Subcutaneous patient-derived xenograft (PDX)	2~4-fold increase in KPC model, ~1.5-fold in PDX compared to without iRGD,	20
Urokinase plasminogen activator receptor (uPAR) targeting peptide modified iron oxide nanoparticles	caveolae mediated	10 nm core	n/a	Orthotopic human MIA PaCa-2 xenograft	3~4-fold increased signal compared to the free peptide	38
Albumin-bound curcumin nanoparticles	caveolar mediated	130-150nm	~ - 20 mV	Subcutaneous human MIA PaCa-2 xenograft	2~10-fold increase at different time points	32
Transferrin conjugated doxorubicin-loaded human serum albumin nanoparticles	caveolae mediated	~ 220 nm	~ - 34.3 mV	*In vitro* human metastatic CAPAN-1 cells	n/a	33
Albumin-bound paclitaxel, ABI-007 (Abraxane)	caveolar mediated	~130 nm	n/a	Subcutaneous human MIA PaCa-2 xenograft	Deeper penetration via intratumoral injection	35
Gemcitabine-loaded albumin nanoparticles	caveolar mediated	~150 nm	~ -10 mV	Subcutaneous human BxPC3 xenograft	n/a	36
γ-glutamyl transpeptidase-responsive camptothecin-polymer conjugate	caveolae mediated	~9 nm	~ -10 mV changed to ~ + 5 mV after γ-glutamyl cleavage	Subcutaneous and orthotopic human BxPC3 xenografts	~2-fold increase compared to non-cleavage polymer	43
